# The Association between Dietary Nutrient Intake and Acceleration of Aging: Evidence from NHANES

**DOI:** 10.3390/nu16111635

**Published:** 2024-05-27

**Authors:** Jianhua Ma, Pingan Li, Yue Jiang, Xinghua Yang, Yanxia Luo, Lixin Tao, Xiuhua Guo, Bo Gao

**Affiliations:** Beijing Municipal Key Laboratory of Clinical Epidemiology, School of Public Health, Capital Medical University, No.10 Xitoutiao, Youanmen Street, Beijing 100069, China

**Keywords:** dietary nutrient intake, acceleration of aging, dietary balance, NHANES

## Abstract

The acceleration of aging is a risk factor for numerous diseases, and diet has been identified as an especially effective anti-aging method. Currently, research on the relationship between dietary nutrient intake and accelerated aging remains limited, with existing studies focusing on the intake of a small number of individual dietary nutrients. Comprehensive research on the single and mixed anti-aging effects of dietary nutrients has not been conducted. This study aimed to comprehensively explore the effects of numerous dietary nutrient intakes, both singly and in combination, on the acceleration of aging. Data for this study were extracted from the 2015–2018 National Health and Nutrition Examination Surveys (NHANES). The acceleration of aging was measured by phenotypic age acceleration. Linear regression (linear), restricted cubic spline (RCS) (nonlinear), and weighted quantile sum (WQS) (mixed effect) models were used to explore the association between dietary nutrient intake and accelerated aging. A total of 4692 participants aged ≥ 20 were included in this study. In fully adjusted models, intakes of 16 nutrients were negatively associated with accelerated aging (protein, vitamin E, vitamin A, beta-carotene, vitamin B1, vitamin B2, vitamin B6, vitamin K, phosphorus, magnesium, iron, zinc, copper, potassium, dietary fiber, and alcohol). Intakes of total sugars, vitamin C, vitamin K, caffeine, and alcohol showed significant nonlinear associations with accelerated aging. Additionally, mixed dietary nutrient intakes were negatively associated with accelerated aging. Single dietary nutrients as well as mixed nutrient intake may mitigate accelerated aging. Moderately increasing the intake of specific dietary nutrients and maintaining dietary balance may be key strategies to prevent accelerated aging.

## 1. Introduction

Aging is defined as the progressive decline in physiological functions that ultimately leads to death [1]. The aging process demonstrates heterogeneity due to inter-individual differences. Chronological age simply indicates the passage of time and does not comprehensively capture the complexity of aging [2]. Consequently, the assessment of aging has become a critical topic. Several methodologies have been proposed, utilizing molecular variables such as the epigenetic clock (expressed as DNA methylation age) [3] and leukocyte telomere length [4]. Nonetheless, indicators based on clinically observable data or phenotypes often provide more reliable aging predictions. In response to this, Levine et al. introduced a novel aging indicator known as phenotypic age [5]. This indicator can distinguish mortality risk among individuals of identical chronological age by utilizing clinical chemical biomarker data from multiple systems. It not only identifies individuals at risk of multiple diseases and mortality but also stratifies populations into varying risk levels, encompassing the healthiest and unhealthiest [6].

Phenotypic age acceleration, which reflects the variance between biological and chronological age, reveals accelerated aging within individuals. Studies have shown that accelerated aging is a significant risk factor for numerous diseases, including heart disease [6], chronic respiratory disease [7], depression [8], cancer [2], and others. Li et al. discovered that accelerated aging could mediate the associations between unhealthy lifestyles and the risk of cardiovascular disease, cancer, and all-cause mortality [9]. Additionally, accelerated aging may result in a deterioration of physiological functions, such as impaired lung function [10]. These findings underscore the broad and detrimental impacts of accelerated aging on overall health, emphasizing the imperative of comprehensive research into the aging process. Notably, interventions through various methods have been shown to decrease the risk of accelerated aging [11].

Among these interventions, diet has been identified as a key factor in regulating the aging process [12]. Specifically, increased consumption of dietary selenium, dietary fiber, and dietary copper could decelerate the aging process [13,14,15]. Foods abundant in flavonoids [16] and dietary antioxidant-rich components [17] have been shown to decrease the risk of accelerated aging. Dietary macronutrients, high-quality carbohydrates, and plant proteins were among the components [18] that may exert protective effects against accelerated aging. Currently, research on the relationship between dietary nutrient intake and accelerated aging remains limited, with existing studies focusing on the intake of a small number of individual dietary nutrients. However, the intake of daily dietary nutrients is complex and varied, and many aspects of it remain underexplored.

In this study, we utilized nationally representative data from adult populations in the United States to explore the effects of numerous dietary nutrient intakes, both singly and in combination, on the acceleration of aging. These dietary nutrients encompassed macronutrients, micronutrients, and other nutritional components (including dietary fiber, caffeine, theobromine, and alcohol). By exploring these dietary nutrients, we seek to propose more comprehensive and effective dietary adjustment strategies for decreasing the risk of accelerated aging.

## 2. Materials and Methods

### 2.1. Study Population

In this study, data were extracted from the publicly available National Health and Nutrition Examination Surveys (NHANES) spanning the years 2015–2018. The NHANES were conducted by the Centers for Disease Control and Prevention (CDC) and comprised a series of cross-sectional surveys, employing a multistage stratified composite design. The NHANES had received approval from the National Center for Health Statistics Research Ethics Review Board, with all participants providing informed consent before their involvement [19].

**Figure 1:**
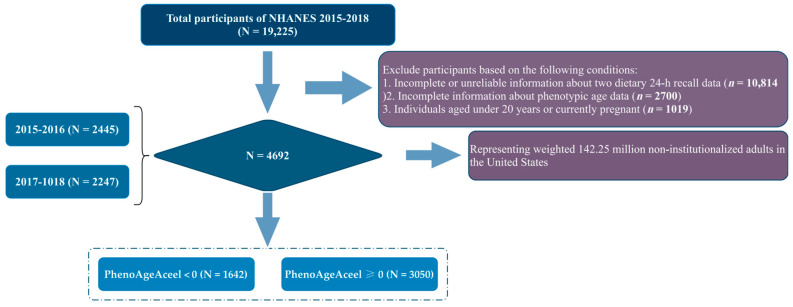
Study flow diagram. NHANES, National Health and Nutritional Examination Surveys; PhenoAgeAceel, phenotypic age acceleration.

Data from two cycles of the NHANES (2015–2018) were utilized in this study, comprising a total of 19,225 participants. Subsequently, individuals with incomplete or unreliable 24-hour dietary recall data (*n* = 10,814), incomplete phenotypic age data (*n* = 2700), and those aged under 20 years old or currently pregnant (*n* = 1019) were excluded from the analysis (Figure 1).

### 2.2. Assessment of Dietary Nutrient Intake

The dietary information in the NHANES was obtained through 24-hour dietary recall interviews conducted in two stages: first at the Mobile Examination Center (MEC) and subsequently via telephone 3–10 days later. To ensure a comprehensive assessment of total dietary intakes, we utilized two consecutive and reliable 24-hour dietary recalls. Dietary intake was defined as the average of the data from these two recalls. In cases where only a single dietary recall was available, that value was used instead of the average [20,21].

We analyzed a total of 38 dietary nutrients derived from the NHANES’s dietary questionnaire, encompassing 8 macronutrients, 17 vitamins, 9 minerals, and 4 other nutritional components. The macronutrients included protein (gm), carbohydrate (gm), total sugars (gm), total fat (gm), cholesterol (mg), total saturated fatty acids (gm), total monounsaturated fatty acids (gm), and total polyunsaturated fatty acids (gm). The vitamins included vitamin E (mg), retinol (mcg), vitamin A (mcg), alpha-carotene (mcg), beta-carotene (mcg), beta-cryptoxanthin (mcg), lycopene (mcg), lutein + zeaxanthin (mcg), vitamin B1 (mg), vitamin B2 (mg), niacin (mg), vitamin B6 (mg), total choline (mg), vitamin B12 (mcg), vitamin C (mg), vitamin D (mcg), and vitamin K (mcg). The minerals included calcium (mg), phosphorus (mg), magnesium (mg), iron (mg), zinc (mg), copper (mg), sodium (mg), potassium (mg), and selenium (mcg). Additionally, the other nutritional components included dietary fiber (gm), caffeine (mg), theobromine (mg), and alcohol (gm).

### 2.3. Assessment of Phenotypic Age and Phenotypic Age Acceleration

Phenotypic age was calculated by 10 aging-related variables: chronological age, albumin (liver), creatinine (kidney), glucose (metabolic), C-reactive protein (inflammation), lymphocyte percent (immune), mean cell volume (immune), red blood cell distribution width (immune), alkaline phosphatase (liver), and white blood cell count (immune). The formula used for computing phenotypic age has been detailed previously [6,8,22].

Phenotypic age acceleration (PhenoAgeAccel) was determined as the residual obtained from a linear regression model where phenotypic age was regressed on chronological age. PhenoAgeAccel reflects the deviation of phenotypic age from chronological age, with positive values indicating accelerated aging and negative values indicating a younger-than-expected phenotype [6,8,22].

In this study, the acceleration of aging was measured by PhenoAgeAccel. The calculations for phenotypic age were conducted using the “BioAge” R package version 0.1.0 [23].

### 2.4. Assessment of Covariates

This study incorporated a range of potential covariates, including age, sex, ethnicity, educational level, exercise, diabetes, history of disease, smoking status, body mass index (BMI), family poverty-income ratio (PIR), drinking status, hyperlipidemia, and hypertension [20,24].

### 2.5. Statistical Analysis

Considering the complex sampling design of the NHANES, we employed two-year dietary sample weights in our analysis. Student’s *t*-test and chi-square tests were employed to evaluate demographic characteristics based on PhenoAgeAccel status (binary). A Mann–Whitney U test was utilized to investigate statistical differences in dietary nutrient intake across different strata of PhenoAgeAccel status (binary).

To address the right-skewed distribution of dietary nutrient intake, we applied log transformation to approximate a normal distribution. Weighted multivariable linear regression was then employed to investigate the associations between individual dietary nutrient intake and the acceleration of aging. To explore potential non-linear associations, we incorporated the restricted cubic spline (RCS) model with 3 knots (10th, 50th, and 90th quartiles). The statistical significance of the non-linear association was determined by *p* overall and *p* non-linear. All analyses were adjusted for confounding variables, including age, sex, ethnicity, educational level, exercise, diabetes, history of disease, smoking status, BMI, family PIR, hyperlipemia, and hypertension.

Subsequently, Spearman’s correlation analysis was conducted to evaluate associations among dietary nutrient intakes. Furthermore, all dietary nutrient intakes were integrated into the weighted quantile sum (WQS) model for 1000 iterations to examine the collective impact of nutrient mixtures on aging. Participants were randomly divided into training (60%) and validation sets for WQS modeling. The “gWQS” R package version 3.0.5 was utilized to calculate the WQS index, representing the weighted sum of individual dietary nutrient intakes ranging from 0 to 1. This index elucidated the cumulative effect on aging attributed to a one-quantile increase in the intake of diverse dietary nutrients [22].

All statistical analyses were performed using R version 4.3.3. A significance threshold of two-sided *p* ≤ 0.05 was employed to determine statistical significance.

## 3. Results

### 3.1. Participant Characteristics

The final sample for analysis included 4692 individuals, representing an estimated 142.3 million non-institutionalized adults in the United States (Figure 1). The overall weighted prevalence of accelerated aging in the total population was 65.0%. The demographic characteristics of participants based on the presence or absence of accelerated aging are presented in Table 1. Significant differences were observed in sociodemographic, lifestyle, and health-related factors regardless of the presence of accelerated aging (*p* < 0.05), except for sex, drinking status, and hyperlipemia (*p* = 0.851, *p* = 0.925, and *p* = 0.077, respectively).

Table 2 presents dietary nutrient intake according to PhenoAgeAceel. Significant differences were observed across the presence or absence of accelerated aging for intakes of protein, cholesterol, vitamin E, vitamin A, alpha-carotene, beta-carotene, lutein + zeaxanthin, vitamin B1, vitamin B2, vitamin B6, vitamin C, vitamin K, calcium, phosphorus, magnesium, iron, zinc, copper, potassium, dietary fiber, and alcohol (*p* < 0.05), while other dietary nutrient intakes showed no difference.

### 3.2. Association between Individual Dietary Nutrient Intake and Accelerated Aging

The results of the weighted multivariable linear regression analysis for associations between individual dietary nutrient intake and accelerated aging are presented in Table 3. Minimally adjusted and fully adjusted models revealed that 16 different variables were significantly and negatively associated with accelerated aging, including 1 macronutrient, 7 vitamins, 6 minerals, and 2 other nutritional components.

In the fully adjusted model, intakes of the following dietary nutrients had potential negative associations with accelerated aging: protein (β: −0.822, 95% CI: −1.439, −0.205, *p* = 0.023), vitamin E (β: −1.023, 95% CI: −1.598, −0.449, *p* = 0.004), vitamin A (β: −0.589, 95% CI: −1.059, −0.120, *p* = 0.030), beta-carotene (β: −0.300, 95% CI: −0.457, −0.143, *p* = 0.003), vitamin B1 (β: −0.693, 95% CI: −1.181, −0.204, *p* = 0.017), vitamin B2 (β: −0.789, 95% CI: −1.303, −0.275, *p* = 0.011), vitamin B6 (β: −0.643, 95% CI: −1.092, −0.194, *p* = 0.016), vitamin K (β: −0.465, 95% CI: −0.791, −0.138, *p* = 0.016), phosphorus (β: −0.894, 95% CI: −1.605, −0.184, *p* = 0.030), magnesium (β: −1.126, 95% CI: −1.913, −0.339, *p* = 0.016), iron (β: −0.919, 95% CI: −1.532, −0.305, *p* = 0.013), zinc (β: −0.860, 95% CI: −1.438, −0.282, *p* = 0.013), copper (β: −1.111, 95% CI: −1.782, −0.439, *p* = 0.007), potassium (β: −0.908, 95% CI: −1.517, −0.298, *p* = 0.013), dietary fiber (β: −1.190, 95% CI: −1.708, −0.672, *p* = 0.001), and alcohol (β: −0.089, 95% CI: −0.148, −0.030, *p* = 0.011).

**Figure 2:**
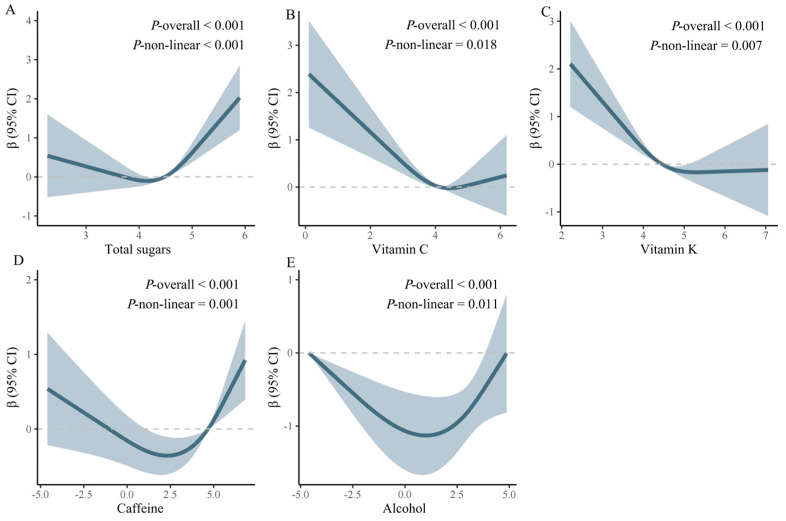
Nonlinear associations between dietary nutrient intake and the acceleration of aging. (**A**): The relationship between total sugar intake and the acceleration of aging (fully adjusted RCS model). The baseline value (β = 0) is represented by a dashed line, with the inflection point occurring at 4.148 (units: log-transformed intake of dietary nutrients). (**B**): The relationship between vitamin C intake and the acceleration of aging (fully adjusted RCS model). The baseline value (β = 0) is represented by a dashed line, with the inflection point occurring at 4.418 (units: log-transformed intake of dietary nutrients). (**C**): The relationship between vitamin K intake and the acceleration of aging (fully adjusted RCS model). The baseline value (β = 0) is represented by a dashed line, with the inflection point occurring at 5.279 (units: log-transformed intake of dietary nutrients). (**D**): The relationship between caffeine intake and the acceleration of aging (fully adjusted RCS model). The baseline value (β = 0) is represented by a dashed line, with the inflection point occurring at 2.285 (units: log-transformed intake of dietary nutrients). (**E**): The relationship between alcohol intake and the acceleration of aging (fully adjusted RCS model). The baseline value (β = 0) is represented by a dashed line, with the inflection point occurring at 1.016 (units: log-transformed intake of dietary nutrients). Dietary nutrient intakes were log-transformed. Adjustments in the model accounted for the following variables: age, sex, ethnicity, educational level, exercise, diabetes, history of disease, smoking status, BMI, family PIR, drinking status, hyperlipemia, and hypertension.

Interestingly, the RCS curve model effectively captured the nonlinear associations between intakes of total sugars, vitamin C, vitamin K, caffeine, alcohol, and accelerated aging (Figure 2). In the fully adjusted model, the associations of these nutrients with accelerated aging changed at these points (total sugars: 4.148; vitamin C: 4.418; vitamin K: 5.279; caffeine: 2.285; and alcohol: 1.016) (units: log-transformed intake of dietary nutrients). Each value of *p* overall and *p* non-linear was less than 0.05. Dose–response relationships between log-transformed intakes of other dietary nutrients and accelerated aging are shown in Figures S1–S4.

**Figure 3:**
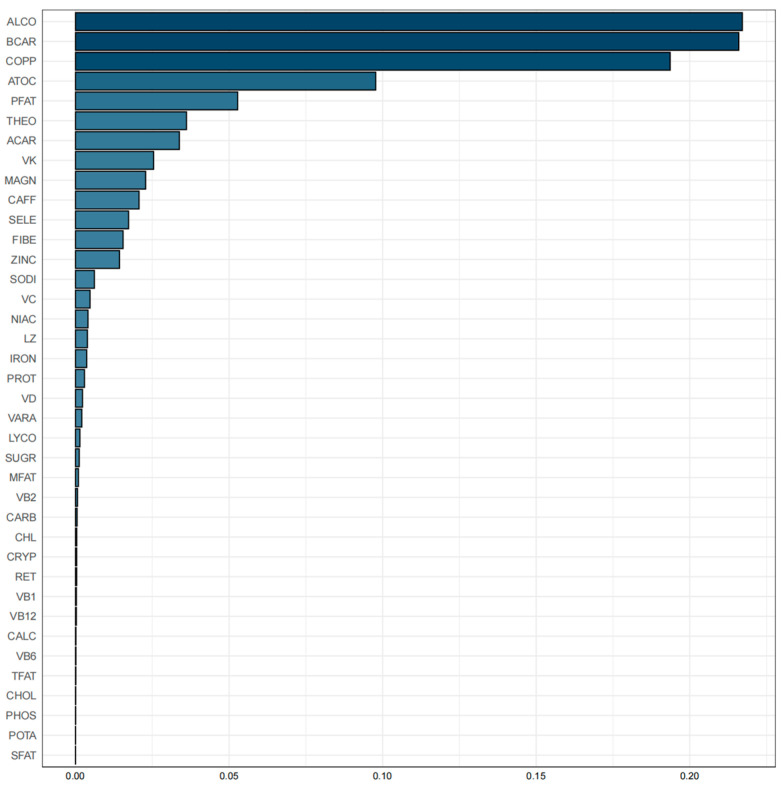
Weighted values of dietary nutrient intakes for the acceleration of aging in WQS models. Abbreviations: ALCO: alcohol, BCAR: beta-carotene, COPP: copper, ATOC: vitamin E, PFAT: total polyunsaturated fatty acids, THEO: theobromine, ACAR: vitamin A, VK: vitamin K, MAGN: magnesium, CAFF: caffeine, SELE: selenium, FIBE: dietary fiber, ZINC: zinc, SODI: sodium, VC: vitamin C, NIAC: niacin, LZ: lutein + zeaxanthin, IRON: iron, PROT: protein, VD: vitamin D, VARA: vitamin A, LYCO: lycopene, SUGR: total sugars, MFAT: total monounsaturated fatty acids, VB2: vitamin B2, CARB: carbohydrate, CHL: total choline, CRYP: beta-cryptoxanthin, RET: retinol, VB1: vitamin B1, VB12: vitamin B12, CALC: calcium, VB6: vitamin B6, TFAT: total fat, CHOL: cholesterol, PHOS: phosphorus, POTA: potassium, SFAT: total saturated fatty acids.

Spearman’s analysis revealed significant positive associations among the majority of 38 dietary nutrients (Figure S5). The WQS index of mixed dietary nutrients log-transformed intakes was negatively associated with accelerated aging (β: −0.611, 95% CI: −1.017, −0.205, *p* = 0.003). Additionally, the top three nutrients with higher weights in the WQS were: alcohol (21.71%), beta-carotene (21.60%), and copper (19.36%) (Figure 3).

Models were adjusted for age, sex, ethnicity, educational level, exercise, diabetes, history of disease, smoking status, BMI, family PIR, drinking status, hyperlipemia, and hypertension.

## 4. Discussion

Before exploring accelerated aging, it is vital to grasp the importance of the associations between dietary nutrients and chronic diseases. Studies have indicated that the intake of dietary minerals such as magnesium, zinc, and selenium is negatively associated with peripheral arterial disease (PAD), hyperuricemia (HUA), and Hashimoto’s thyroiditis [25,26,27]. Additionally, specific nutrients such as caffeine, folic acid, selenium, and magnesium have exhibited protective properties against depression [28]. Furthermore, numerous studies have provided evidence that the intake of certain dietary nutrients can reduce the risk of some cancers [21,29,30]. Notably, accelerated aging plays a crucial role in the development of numerous chronic diseases.

Several studies have explored the association between dietary nutrient intake and accelerated aging. Intakes of dietary selenium, dietary fiber, and dietary copper have been shown to reduce the risk of accelerated aging [13,14,15]. Additionally, dietary macronutrients and high-quality carbohydrates also exhibited a protective effect against aging [18]. However, existing studies have primarily focused on individual nutrients or a limited number of nutrients, necessitating further research for a more comprehensive understanding of the associations between dietary nutrients and accelerated aging. Considering the diversity of daily diets, there are numerous nutrients with anti-aging properties that remain to be fully investigated. Accordingly, our study comprehensively incorporated a variety of dietary nutrients commonly consumed in the daily diet, aiming to elucidate a more comprehensive anti-aging dietary strategy.

Our study identified linear negative associations between intakes of 16 dietary nutrients and accelerated aging. These nutrients included 1 macronutrient (protein), 7 vitamins (vitamin E, vitamin A, beta-carotene, vitamin B1, vitamin B2, vitamin B6, and vitamin K), 6 minerals (phosphorus, magnesium, iron, zinc, copper, and potassium), and 2 other nutritional components (dietary fiber and alcohol). In addition, intakes of total sugars, vitamin C, vitamin K, caffeine, and alcohol showed significant nonlinear associations with accelerated aging. Furthermore, mixed dietary nutrient intakes were negatively associated with accelerated aging.

Animal experiments have shown that excessive consumption of pro-inflammatory diets, such as high-fat diets, may result in abnormal expression of aging-related genes, which in turn accelerates aging [31]. The mechanisms by which various nutrients affect aging may be through their role in cellular functions, including DNA repair and chromosome maintenance, inflammation, oxidative stress, and DNA methylation [32].

Our study found that intakes of dietary copper, iron, magnesium, and zinc were negatively associated with accelerated aging. The anti-aging effects of copper and iron may be related to their antioxidant capacity [13,33]. Magnesium is essential for stabilizing DNA, protecting cells from ROS damage, and promoting DNA replication and transcription. Its deficiency can induce genomic instability, alter DNA repair, and reduce mitochondrial function, ultimately accelerating cellular senescence and aging [34]. Additionally, zinc may decelerate the accelerated aging process by affecting the cellular autophagy pathway. It also plays a critical role in various age-related metabolic changes, including oxidative stress, impaired energy metabolism, and chronic inflammatory responses [35].

We also observed that vitamin B2, vitamin A, vitamin E, and beta-carotene were negatively associated with accelerated aging. These vitamins exert anti-aging effects through their antioxidant effects or anti-inflammatory effects [17,36]. In addition, we found a negative association between vitamin B6 and accelerated aging as well, which is consistent with previous findings [37]. Research has indicated that low vitamin B6 levels are associated with muscle wasting, weakness, and overall mortality. Moreover, vitamin B6 supplements have been shown to reduce diabetes complications, reduce cognitive aging, and prevent coronary heart disease [38,39]. These findings further support its potential role in preventing aging, but the exact mechanism remains unclear.

Furthermore, our study found that dietary fiber may reduce the risk of accelerated aging, possibly due to its antioxidant effects or inhibition of inflammation [15]. Research has shown that characteristic dietary fiber compounds have a beneficial effect on the cognitive abilities of aging mice, improving both learning and memory functions while enhancing antioxidant defenses and reducing inflammation. This underscores the potential anti-aging efficacy of dietary fiber complexes in naturally aging mice [40]. Additionally, our research found intakes of phosphorus, protein, potassium, and vitamin B1 were also negatively associated with accelerated aging. While there was no direct evidence to validate the association between these nutrients and accelerated aging, studies have emphasized the potential role of these nutrients in the prevention of related diseases such as sarcopenia, rheumatoid arthritis, cancer, and others [21,41,42,43,44,45,46,47].

We identified that intakes of alcohol, caffeine, total sugars, vitamin C, and vitamin K had significant nonlinear associations with accelerated aging. Previous studies have suggested a positive association between alcohol consumption and accelerated aging [48,49,50]. However, our study unexpectedly indicated that moderate alcohol intake might decelerate aging. Further investigation is needed to explore the potential mechanisms of this association. In addition, previous research has reported that caffeine, total dietary sugar, vitamin C, and vitamin K can each decelerate aging through distinct mechanisms [51,52,53,54,55]. Our study observed a gradual decrease in the risk of accelerated aging with increasing intakes of these nutrients. However, this trend may be reversed with high intake levels, potentially leading to an increased risk of aging. Currently, there is no research explicitly elucidating the biological mechanisms behind how excessive caffeine consumption accelerates aging. However, studies suggest that excessive caffeine intake can affect psychological health, suggesting potential risks associated with overconsumption [56]. Additionally, excessive total sugar intake may lead to protein glycosylation, oxidative stress, inflammation, insulin resistance, and dyslipidemia, all characteristic changes associated with aging [57]. Further in-depth investigation is necessary to understand how excessive intake of vitamin C and vitamin E may also accelerate aging. Moreover, our study revealed a negative association between mixed nutrient intake and accelerated aging. The finding underscored the significance of maintaining a balanced diet by focusing on the moderate intake of a variety of dietary nutrients. It emphasized the need to increase the intake of nutrients that are beneficial in combating aging while still maintaining moderation. Increasing the intake of protein-rich foods like soy and milk, as well as incorporating green leafy vegetables, fruits, beans, and nuts, which are abundant in fiber and various vitamins, and moderately consuming coffee, can form a comprehensive dietary strategy with potential anti-aging benefits.

### 4.1. Strengths

Our study included a large number of dietary nutrients and analyzed the associations between dietary nutrients and accelerated aging more comprehensively in a United States population. We provided a comprehensive view of the linear, nonlinear, and mixed effects between these nutrients and accelerated aging.

### 4.2. Limitations

Firstly, our study utilized a cross-sectional design and therefore could not determine a causal association between dietary nutrients and accelerated aging. Future prospective or causal extrapolation studies are needed for validation. Secondly, the definition of phenotypic age was based on chronological age and several biomarkers, which may contribute to the risk of over-adjustment for confounders in the regression model. However, we have attempted to mitigate this problem through multiple collinearity tests. Finally, we used a 24-hour dietary recall to assess dietary nutrient intake and utilized telephone interviews, which may be subject to recall bias and have limitations in producing conclusive assessments.

## 5. Conclusions

This study revealed that intakes of 1 macronutrient (protein), 7 vitamins (vitamin E, vitamin A, beta-carotene, vitamin B1, vitamin B2, vitamin B6, and vitamin K), 6 minerals (phosphorus, magnesium, iron, zinc, copper, and potassium), and 2 nutritional components (dietary fiber and alcohol) were negatively associated with accelerated aging. In addition, total sugars, vitamin C, vitamin K, caffeine, and alcohol showed significant nonlinear associations with accelerated aging. Furthermore, mixed dietary nutrient intakes were negatively associated with accelerated aging. Therefore, a moderate increase in the intake of specific dietary nutrients and the maintenance of dietary balance may emerge as pivotal strategies in the prevention of accelerated aging. However, further prospective studies are warranted to elucidate our findings.

## Figures and Tables

**Table 1:** Baseline characteristics according to PhenoAgeAceel.

	PhenoAgeAceel < 0 Unweighted Sample Size (%) or Mean (SD) ^a^	PhenoAgeAceel ≥ 0 Unweighted Sample Size (%) or Mean (SD) ^a^	*p*-Value ^b^
All participants	1642 (35.0)	3050 (65.0)	
Age	48.2 (17.5)	50.0 (17.3)	0.044
Sex			
Female	789 (49.3)	1511 (49.6)	0.851
Male	853 (50.7)	1539 (50.4)	
Ethnicity			
White	654 (70.5)	1270 (67.8)	
Black	267 (6.7)	585 (8.7)	0.010
Mexican	190 (6.0)	480 (9.0)	
Other	531 (16.8)	715 (14.6)	
Educational level			
Pre-high school	260 (8.2)	586 (11.5)	<0.001
Post-high school	1076 (75.6)	1706 (61.7)	
High school	305 (16.2)	754 (26.8)	
Exercise			
No	642 (28.3)	1707 (49.7)	<0.001
Yes	999 (71.7)	1337 (50.3)	
Diabetes			
No	1527 (95.1)	2405 (83.5)	<0.001
Yes	115 (4.9)	643 (16.5)	
History of disease			
No	706 (44.0)	819 (27.5)	<0.001
Yes	933 (56.0)	2225 (72.5)	
Smoking status			
No	1087 (67.9)	1612 (52.3)	<0.001
Yes	554 (32.1)	1435 (47.7)	
BMI (kg/m^2^)			
25 ≤ BMI < 30	605 (36.2)	927 (28.9)	<0.001
30 ≤ BMI	333 (21.7)	1564 (53.6)	
BMI < 25	696 (42.2)	529 (17.6)	
Family PIR			
<100%	1284 (91.9)	2210 (86.9)	<0.001
>100%	205 (8.1)	548 (13.1)	
Drinking status			
No	208 (11.6)	379(11.8)	0.925
Yes	1121 (88.4)	1980(88.2)	
Hyperlipemia			
No	1070 (68.1)	1859 (63.8)	0.077
Yes	565 (31.9)	1163 (36.2)	
Hypertension			
No	1174 (74.8)	1735 (61.9)	<0.001
Yes	468 (25.2)	1307 (38.1)	
PhenoAge	45.0 (17.7)	55.7 (18.2)	<0.001
PhenoAgeAceel	−3.1 (2.3)	5.7 (4.8)	<0.001

Abbreviations: SD, standard deviation; PIR, poverty income ratio; BMI, body mass index. ^a^ Weighted percentage or mean (SD); ^b^ *p*-values consider complex sampling.

**Table 2:** Dietary nutrient intake according to PhenoAgeAceel.

	PhenoAgeAceel < 0 Mean (SD) ^a^	PhenoAgeAceel ≥ 0 Mean (SD) ^a^	*p*-Value ^b^
Macronutrients			
Protein (gm)	80.4 [60.3, 102.1]	75.9 [58.2, 99.4]	0.003
Carbohydrate (gm)	233.0 [174.1, 293.3]	224.6 [168.3, 291.2]	0.165
Total sugars (gm)	88.9 [60.3, 125.0]	90.4 [59.9, 130.6]	0.419
Total fat (gm)	79.9 [58.9, 103.1]	78.1 [58.2, 103.5]	0.549
Cholesterol (mg)	248.2 [162.0, 364.0]	268.0 [173.0, 402.0]	0.029
Total saturated fatty acids (gm)	24.9 [17.9, 34.5]	25.6 [18.1, 34.7]	0.529
Total monounsaturated fatty acids (gm)	27.8 [20.3, 37.0]	26.5 [19.3, 35.7]	0.194
Total polyunsaturated fatty acids (gm)	18.4 [13.1, 23.7]	17.6 [12.4, 24.6]	0.428
Vitamins			
Vitamin E (mg)	8.7 [6.1, 12.2]	7.7 [5.4, 10.9]	<0.001
Retinol (mcg)	372.7 [224.1, 567.4]	360.0 [223.0, 546.2]	0.478
Vitamin A (mcg)	611.5 [399.9, 896.0]	530.2 [352.0, 791.3]	<0.001
Alpha-carotene (mcg)	122.0 [38.5, 624.5]	73.5 [22.5, 375.0]	<0.001
Beta-carotene (mcg)	1650.2 [666.3, 3811.3]	1112.2 [453.2, 2559.2]	<0.001
Beta-cryptoxanthin (mcg)	43.3 [18.5, 104.5]	41.5 [16.0, 92.9]	0.064
Lycopene (mcg)	2634.0 [866.5, 6716.5]	2568.8 [810.0, 6761.0]	0.469
Lutein + zeaxanthin (mcg)	1076.5 [599.6, 2233.7]	900.5 [510.0, 1719.0]	<0.001
Vitamin B1 (mg)	1.6 [1.1, 2.0]	1.4 [1.1, 1.9]	0.003
Vitamin B2 (mg)	2.0 [1.5, 2.7]	1.9 [1.4, 2.5]	0.005
Niacin (mg)	24.5 [18.0, 32.5]	23.8 [17.5, 30.7]	0.057
Vitamin B6 (mg)	1.9 [1.4, 2.7]	1.8 [1.3, 2.5]	<0.001
Total choline (mg)	312.4 [230.8, 415.2]	316.0 [227.3, 416.8]	0.806
Vitamin B12 (mcg)	3.9 [2.6, 6.2]	4.0 [2.6, 6.0]	0.698
Vitamin C (mg)	73.5 [35.1, 123.6]	55.5 [28.0, 100.5]	<0.001
Vitamin D (mcg)	3.6 [2.0, 6.1]	3.5 [1.9, 5.8]	0.139
Vitamin K (mcg)	101.4 [61.1, 170.0]	84.6 [51.6, 143.2]	<0.001
Minerals			
Calcium (mg)	913.2 [658.5, 1246.4]	875.5 [623.9, 1169.4]	0.009
Phosphorus (mg)	1335.5 [1060.8, 1729.2]	1299.7 [1007.5, 1640.8]	0.005
Magnesium (mg)	310.1 [238.8, 403.5]	279.0 [210.5, 355.5]	<0.001
Iron (mg)	13.7 [10.2, 18.2]	12.8 [9.7, 16.9]	0.001
Zinc (mg)	10.5 [7.9, 14.5]	10.0 [7.3, 13.6]	0.001
Copper (mg)	1.2 [0.9, 1.6]	1.1 [0.8, 1.4]	<0.001
Sodium (mg)	3351.6 [2489.1, 4319.0]	3255.9 [2474.4, 4148.6]	0.397
Potassium (mg)	2669.0 [2077.3, 3392.3]	2493.0 [1916.4, 3120.0]	<0.001
Selenium (mcg)	110.0 [80.7, 144.3]	107.9 [78.0, 141.1]	0.099
Other nutritional components			
Dietary fiber (gm)	17.6 [12.3, 24.4]	15.1 [10.7, 21.0]	<0.001
Caffeine (mg)	123.5 [47.5, 236.8]	139.5 [48.0, 252.1]	0.245
Theobromine (mg)	13.4 [0.0, 41.0]	14.9 [0.0, 44.5]	0.707
Alcohol (gm)	0.0 [0.0, 12.9]	0.0 [0.0, 5.6]	<0.001

Abbreviations: SD, standard deviation. ^a^ Weighted percentage or mean (SD); ^b^ *p*-values consider complex sampling.

**Table 3:** Linear regression analyses of associations between dietary nutrient intake and acceleration of aging.

Variable	Model 1		Model 2	
	Β (95% CI)	*p*-Value	Β (95% CI)	*p*-Value
Macronutrients				
Protein	−1.143 (−1.854, −0.432)	0.004	−0.822 (−1.439, −0.205)	0.023
Carbohydrate	−0.206 (−0.758, 0.346)	0.472	0.006 (−0.610, 0.623)	0.984
Total sugars	0.323 (−0.113, 0.760)	0.160	0.479 (0.037, 0.920)	0.055
Total fat	−0.323 (−1.122, 0.476)	0.436	−0.530 (−1.208, 0.147)	0.151
Cholesterol	0.361 (−0.018, 0.740)	0.074	0.054 (−0.320, 0.427)	0.783
Total saturated fatty acids	0.236 (−0.453, 0.925)	0.508	−0.234 (−0.867, 0.399)	0.483
Total monounsaturated fatty acids	−0.576 (−1.299, 0.147)	0.131	−0.626 (−1.222, −0.030)	0.062
Total polyunsaturated fatty acids	−0.545 (−1.171, 0.080)	0.100	−0.482 (−1.045, 0.080)	0.119
Vitamins				
Vitamin E	−1.662 (−2.314, −1.010)	<0.001	−1.023 (−1.598, −0.449)	0.004
Retinol	−0.295 (−0.873, 0.284)	0.328	−0.296 (−0.824, 0.232)	0.294
Vitamin A	−0.900 (−1.492, −0.308)	0.007	−0.589 (−1.059, −0.120)	0.030
Alpha-carotene	−0.296 (−0.439, −0.154)	<0.001	−0.050 (−0.163, 0.062)	0.399
Beta-carotene	−0.696 (−0.921, −0.471)	<0.001	−0.300 (−0.457, −0.143)	0.003
Beta-cryptoxanthin	−0.131 (−0.250, −0.013)	0.040	0.025 (−0.115, 0.164)	0.736
Lycopene	−0.014 (−0.071, 0.044)	0.647	0.005 (−0.056, 0.066)	0.872
Lutein + zeaxanthin	−0.791 (−1.038, −0.544)	<0.001	−0.217 (−0.429, −0.006)	0.067
Vitamin B1	−1.121 (−1.638, −0.603)	<0.001	−0.693 (−1.181, −0.204)	0.017
Vitamin B2	−1.143 (−1.783, −0.504)	0.002	−0.789 (−1.303, −0.275)	0.011
Niacin	−0.727 (−1.349, −0.105)	0.031	−0.462 (−1.035, 0.111)	0.140
Vitamin B6	−0.974 (−1.464, −0.483)	0.001	−0.643 (−1.092, −0.194)	0.016
Total choline	−0.562 (−1.176, 0.053)	0.086	−0.462 (−0.997, 0.073)	0.116
Vitamin B12	−0.197 (−0.548, 0.154)	0.281	−0.300 (−0.710, 0.111)	0.178
Vitamin C	−0.857 (−1.135, −0.579)	<0.001	−0.334 (−0.651, −0.016)	0.062
Vitamin D	−0.197 (−0.419, 0.025)	0.094	−0.044 (−0.254, 0.166)	0.689
Vitamin K	−1.106 (−1.461, −0.751)	<0.001	−0.465 (−0.791, −0.138)	0.016
Minerals				
Calcium	−0.702 (−1.243, −0.161)	0.018	−0.478 (−0.959, 0.002)	0.075
Phosphorus	−1.432 (−2.251, −0.614)	0.002	−0.894 (−1.605, −0.184)	0.030
Magnesium	−2.444 (−3.201, −1.686)	<0.001	−1.126 (−1.913, −0.339)	0.016
Iron	−1.280 (−1.911, −0.648)	0.001	−0.919 (−1.532, −0.305)	0.013
Zinc	−1.078 (−1.660, −0.496)	0.001	−0.860 (−1.438, −0.282)	0.013
Copper	−2.125 (−2.940, −1.311)	<0.001	−1.111 (−1.782, −0.439)	0.007
Sodium	−0.175 (−0.856, 0.506)	0.619	−0.292 (−0.862, 0.279)	0.336
Potassium	−2.034 (−2.728, −1.341)	<0.001	−0.908 (−1.517, −0.298)	0.013
Selenium	−0.587 (−1.185, 0.010)	0.066	−0.428 (−0.980, 0.123)	0.154
Other nutritional components				
Dietary fiber	−2.068 (−2.711, −1.426)	<0.001	−1.190 (−1.708, −0.672)	0.001
Caffeine	0.067 (−0.057, 0.191)	0.299	0.012 (−0.092, 0.115)	0.830
Theobromine	−0.041 (−0.115, 0.033)	0.286	0.006 (−0.060, 0.071)	0.870
Alcohol	−0.166 (−0.227, −0.105)	<0.001	−0.089 (−0.148, −0.030)	0.011

Abbreviations: SD, standard deviation; PIR, poverty income ratio; BMI, body mass index; CI, confidence interval. Dietary nutrient intakes were log-transformed. Model 1 adjusted for age, sex, and ethnicity; Model 2 adjusted for Model 1 plus educational level, exercise, diabetes, history of disease, smoking status, BMI, family PIR, drinking status, hyperlipemia, and hypertension.

## Data Availability

The data supporting the findings of this study are publicly available from the website at https://www.cdc.gov/nchs/nhanes/index.htm, accessed on 1 March 2024.

## Supplementary Materials

The following supporting information can be downloaded at: https://www.mdpi.com/article/10.3390/nu16111635/s1, Figure S1: Dose–response relationships between dietary nutrient intake and accelerated aging (A); Figure S2: Dose–response relationships between dietary nutrient intake and accelerated aging (B); Figure S3: Dose–response relationships between dietary nutrient intake and accelerated aging (C); Figure S4: Dose–response relationships between dietary nutrient intake and accelerated aging (D); Figure S5: Spearman’s correlation matrix among log-transformed dietary nutrient intakes in the study population.

## References

[1] Kanasi E., Ayilavarapu S., Jones J. (2016). The aging population: Demographics and the biology of aging. Periodontology 2000.

[2] Ma Z., Zhu C., Wang H., Ji M., Huang Y., Wei X., Zhang J., Wang Y., Yin R., Dai J. (2023). Association between biological aging and lung cancer risk: Cohort study and Mendelian randomization analysis. iScience.

[3] Horvath S. (2013). DNA methylation age of human tissues and cell types. Genome Biol..

[4] Benetos A., Okuda K., Lajemi M., Kimura M., Thomas F., Skurnick J., Labat C., Bean K., Aviv A. (2001). Telomere length as an indicator of biological aging: The gender effect and relation with pulse pressure and pulse wave velocity. Hypertension.

[5] Levine M.E., Lu A.T., Quach A., Chen B.H., Assimes T.L., Bandinelli S., Hou L., Baccarelli A.A., Stewart J.D., Li Y. (2018). An epigenetic biomarker of aging for lifespan and healthspan. Aging (Albany N. Y.).

[6] Liu Z., Kuo P.L., Horvath S., Crimmins E., Ferrucci L., Levine M. (2018). A new aging measure captures morbidity and mortality risk across diverse subpopulations from NHANES IV: A cohort study. PLoS Med..

[7] Wang T., Duan W., Jia X., Huang X., Liu Y., Meng F., Ni C. (2024). Associations of combined phenotypic ageing and genetic risk with incidence of chronic respiratory diseases in the UK Biobank: A prospective cohort study. Eur. Respir. J..

[8] Gao X., Geng T., Jiang M., Huang N., Zheng Y., Belsky D.W., Huang T. (2023). Accelerated biological aging and risk of depression and anxiety: Evidence from 424,299 UK Biobank participants. Nat. Commun..

[9] Li X., Cao X., Zhang J., Fu J., Mohedaner M., Danzengzhuoga, Sun X., Yang G., Yang Z., Kuo C.L. (2024). Accelerated aging mediates the associations of unhealthy lifestyles with cardiovascular disease, cancer, and mortality. J. Am. Geriatr. Soc..

[10] Ruan Z., Li D., Huang D., Liang M., Xu Y., Qiu Z., Chen X. (2023). Relationship between an ageing measure and chronic obstructive pulmonary disease, lung function: A cross-sectional study of NHANES, 2007–2010. BMJ Open.

[11] Campisi J., Kapahi P., Lithgow G.J., Melov S., Newman J.C., Verdin E. (2019). From discoveries in ageing research to therapeutics for healthy ageing. Nature.

[12] Partridge L., Deelen J., Slagboom P.E. (2018). Facing up to the global challenges of ageing. Nature.

[13] Gong H., Yu Q., Yuan M., Jiang Y., Wang J., Huang P., Zhou J. (2022). The Relationship between Dietary Copper intake and Telomere Length in Hypertension. J. Nutr. Health Aging.

[14] Shu Y., Wu M., Yang S., Wang Y., Li H. (2020). Association of dietary selenium intake with telomere length in middle-aged and older adults. Clin. Nutr..

[15] Tucker L.A. (2018). Dietary Fiber and Telomere Length in 5674 U.S. Adults: An NHANES Study of Biological Aging. Nutrients.

[16] Xing W., Gao W., Zhao Z., Xu X., Bu H., Su H., Mao G., Chen J. (2023). Dietary flavonoids intake contributes to delay biological aging process: Analysis from NHANES dataset. J. Transl. Med..

[17] He H., Chen X., Ding Y., Chen X., He X. (2024). Composite dietary antioxidant index associated with delayed biological aging: A population-based study. Aging (Albany N. Y.).

[18] Zhu X., Xue J., Maimaitituerxun R., Xu H., Zhou Q., Zhou Q., Dai W., Chen W. (2024). Relationship between dietary macronutrients intake and biological aging: A cross-sectional analysis of NHANES data. Eur. J. Nutr..

[19] Cheng T.D., Ferderber C., Kinder B., Wei Y.J. (2023). Trends in Dietary Vitamin A Intake Among US Adults by Race and Ethnicity, 2003–2018. JAMA.

[20] Chen S., Liu J., Kang X., Cui K., Zhang D. (2023). Association of dietary mineral mixture with depressive symptoms: A combination of Bayesian approaches. Prev. Med..

[21] Zhu G., Li Z., Tang L., Shen M., Zhou Z., Wei Y., Zhao Y., Bai S., Song L. (2022). Associations of Dietary Intakes with Gynecological Cancers: Findings from a Cross-Sectional Study. Nutrients.

[22] Chen L., Zhao Y., Liu F., Chen H., Tan T., Yao P., Tang Y. (2022). Biological aging mediates the associations between urinary metals and osteoarthritis among U.S. adults. BMC Med..

[23] Kwon D., Belsky D.W. (2021). A toolkit for quantification of biological age from blood chemistry and organ function test data: BioAge. Geroscience.

[24] Iranpour S., Sabour S. (2019). Inverse association between caffeine intake and depressive symptoms in US adults: Data from National Health and Nutrition Examination Survey (NHANES) 2005–2006. Psychiatry Res..

[25] Wu Z., Ruan Z., Liang G., Wang X., Wu J., Wang B. (2023). Association between dietary magnesium intake and peripheral arterial disease: Results from NHANES 1999–2004. PLoS ONE.

[26] Zhang Y., Liu Y., Qiu H. (2018). Association between Dietary Zinc Intake and Hyperuricemia among Adults in the United States. Nutrients.

[27] Zheng G., Cai Y., Guo Y., Song F., Hu Y., Li L., Zhu L. (2023). The association between dietary selenium intake and Hashimoto’s thyroiditis among US adults: National Health and Nutrition Examination Survey (NHANES), 2007–2012. J. Endocrinol. Investig..

[28] Ekinci G.N., Sanlier N. (2023). The relationship between nutrition and depression in the life process: A mini-review. Exp. Gerontol..

[29] McEligot A.J., Poynor V., Sharma R., Panangadan A. (2020). Logistic LASSO Regression for Dietary Intakes and Breast Cancer. Nutrients.

[30] Yong L.C., Brown C.C., Schatzkin A., Dresser C.M., Slesinski M.J., Cox C.S., Taylor P.R. (1997). Intake of vitamins E, C, and A and risk of lung cancer. The NHANES I epidemiologic followup study. First National Health and Nutrition Examination Survey. Am. J. Epidemiol..

[31] Wang X., Sarker S.K., Cheng L., Dang K., Hu J., Pan S., Zhang J., Xu X., Li Y. (2024). Association of dietary inflammatory potential, dietary oxidative balance score and biological aging. Clin. Nutr..

[32] Paul L. (2011). Diet, nutrition and telomere length. J. Nutr. Biochem..

[33] Arruda L.F., Arruda S.F., Campos N.A., de Valencia F.F., Siqueira E.M. (2013). Dietary iron concentration may influence aging process by altering oxidative stress in tissues of adult rats. PLoS ONE.

[34] Barbagallo M., Veronese N., Dominguez L.J. (2021). Magnesium in Aging, Health and Diseases. Nutrients.

[35] Sun R., Wang J., Feng J., Cao B. (2022). Zinc in Cognitive Impairment and Aging. Biomolecules.

[36] Suwannasom N., Kao I., Pruß A., Georgieva R., Bäumler H. (2020). Riboflavin: The Health Benefits of a Forgotten Natural Vitamin. Int. J. Mol. Sci..

[37] Nwanaji-Enwerem J.C., Colicino E., Gao X., Wang C., Vokonas P., Boyer E.W., Baccarelli A.A., Schwartz J. (2021). Associations of Plasma Folate and Vitamin B6 With Blood DNA Methylation Age: An Analysis of One-Carbon Metabolites in the VA Normative Aging Study. J. Gerontol. A Biol. Sci. Med. Sci..

[38] Kato N., Kimoto A., Zhang P., Bumrungkit C., Karunaratne S., Yanaka N., Kumrungsee T. (2024). Relationship of Low Vitamin B6 Status with Sarcopenia, Frailty, and Mortality: A Narrative Review. Nutrients.

[39] Kannan K., Jain S.K. (2004). Effect of vitamin B6 on oxygen radicals, mitochondrial membrane potential, and lipid peroxidation in H2O2-treated U937 monocytes. Free Radic. Biol. Med..

[40] Yu X., Liang X., Han K., Shi F., Meng N., Li Q. (2022). Anti-Aging Effect of Dietary Fiber Compound Mediated by Guangxi Longevity Dietary Pattern on Natural Aging Mice. Nutrients.

[41] Chen Y.Y., Chen Y.J. (2022). Association between Dietary Calcium and Potassium and Diabetic Retinopathy: A Cross-Sectional Retrospective Study. Nutrients.

[42] Fang J., Cao T., Liu C., Wang D., Zhang H., Tong J., Lin Z. (2023). Association between magnesium, copper, and potassium intakes with risk of rheumatoid arthritis: A cross-sectional study from National Health and Nutrition Examination Survey (NHANES). BMC Public Health.

[43] Leitão C., Mignano A., Estrela M., Fardilha M., Figueiras A., Roque F., Herdeiro M.T. (2022). The Effect of Nutrition on Aging-A Systematic Review Focusing on Aging-Related Biomarkers. Nutrients.

[44] Liu D., Ke Z., Luo J. (2017). Thiamine Deficiency and Neurodegeneration: The Interplay Among Oxidative Stress, Endoplasmic Reticulum Stress, and Autophagy. Mol. Neurobiol..

[45] Rogeri P.S., Zanella R., Martins G.L., Garcia M.D.A., Leite G., Lugaresi R., Gasparini S.O., Sperandio G.A., Ferreira L.H.B., Souza-Junior T.P. (2021). Strategies to Prevent Sarcopenia in the Aging Process: Role of Protein Intake and Exercise. Nutrients.

[46] Strasser B., Volaklis K., Fuchs D., Burtscher M. (2018). Role of Dietary Protein and Muscular Fitness on Longevity and Aging. Aging Dis..

[47] van Dronkelaar C., van Velzen A., Abdelrazek M., van der Steen A., Weijs P.J.M., Tieland M. (2018). Minerals and Sarcopenia; The Role of Calcium, Iron, Magnesium, Phosphorus, Potassium, Selenium, Sodium, and Zinc on Muscle Mass, Muscle Strength, and Physical Performance in Older Adults: A Systematic Review. J. Am. Med. Dir. Assoc..

[48] Kim K., Zheng Y., Joyce B.T., Jiang H., Greenland P., Jacobs D.R., Zhang K., Liu L., Allen N.B., Wilkins J.T. (2022). Relative contributions of six lifestyle- and health-related exposures to epigenetic aging: The Coronary Artery Risk Development in Young Adults (CARDIA) Study. Clin. Epigenet..

[49] Nannini D.R., Joyce B.T., Zheng Y., Gao T., Wang J., Liu L., Jacobs D.R., Schreiner P.J., Liu C., Dai Q. (2023). Alcohol consumption and epigenetic age acceleration in young adults. Aging (Albany N. Y.).

[50] Wang M., Li Y., Lai M., Nannini D.R., Hou L., Joehanes R., Huan T., Levy D., Ma J., Liu C. (2023). Alcohol consumption and epigenetic age acceleration across human adulthood. Aging (Albany N. Y.).

[51] Monacelli F., Acquarone E., Giannotti C., Borghi R., Nencioni A. (2017). Vitamin C, Aging and Alzheimer’s Disease. Nutrients.

[52] Ostojic S.M., Hillesund E.R., Øverby N.C., Vik F.N., Medin A.C. (2023). Individual nutrients and serum klotho levels in adults aged 40–79 years. Food Sci. Nutr..

[53] Popa D.S., Bigman G., Rusu M.E. (2021). The Role of Vitamin K in Humans: Implication in Aging and Age-Associated Diseases. Antioxidants.

[54] Takahashi K., Ishigami A. (2017). Anti-aging effects of coffee. Aging (Albany N. Y.).

[55] Tao L., Zhang W., Zhang Y., Zhang M., Zhang Y., Niu X., Zhao Q., Liu Z., Li Y., Diao A. (2021). Caffeine promotes the expression of telomerase reverse transcriptase to regulate cellular senescence and aging. Food Funct..

[56] Abdoli F., Davoudi M., Momeni F., Djafari F., Dolatshahi B., Hosseinzadeh S., Aliyaki H., Khalili Z. (2024). Estimate the prevalence of daily caffeine consumption, caffeine use disorder, caffeine withdrawal and perceived harm in Iran: A cross-sectional study. Sci. Rep..

[57] Huang C., Liang Z., Ma J., Hu D., Yao F., Qin P. (2023). Total sugar, added sugar, fructose, and sucrose intake and all-cause, cardiovascular, and cancer mortality: A systematic review and dose-response meta-analysis of prospective cohort studies. Nutrition.

