# Supplementary material for: The Association between Dietary Nutrient Intake and Acceleration of Aging: Evidence from NHANES
Source: Nutrients. 2024 May 27;16(11):1635. doi: 10.3390/nu16111635 (PMC11174358; doi:10.3390/nu16111635)
Supplement: Supplementary file 1 [file nutrients-16-01635-s001.zip › nutrients-3008585-supplementary.pdf]

Supplemental Materials

The Association between Dietary Nutrient Intake and Acceleration of Aging: Evidence from NHANES

Figure S1 Dose-response relationships between dietary nutrient intake and accelerated aging (A)

Figure S2 Dose-response relationships between dietary nutrient intake and accelerated aging (B)

Figure S3 Dose-response relationships between dietary nutrient intake and accelerated aging (C)

Figure S4 Dose-response relationships between dietary nutrient intake and accelerated aging (D)

Figure S5 Spearman's correlation matrix among Log-transformed dietary nutrient intakes in the study population

Supplemental Figures

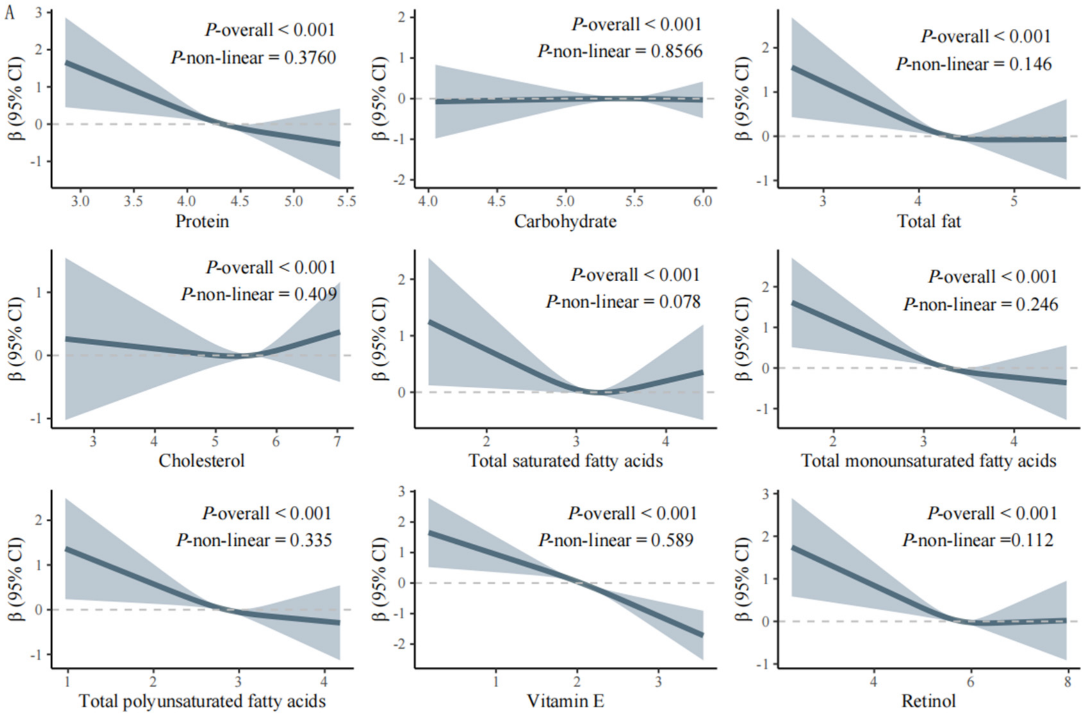

Figure S1 Dose-response relationships between dietary nutrient intake and accelerated aging (A)

The dose-response relationships between dietary nutrient intake (including protein, carbohydrate, total fat, cholesterol, total saturated fatty acids, total monounsaturated fatty acids, total polyunsaturated fatty acids, vitamin E, retinol) and acceleration of aging (fully adjusted RCS Model).

Dietary nutrient intakes were log-transformed. Adjustments in the model accounted for the following variables: age, sex, ethnicity, educational level, exercise, diabetes, history of disease, smoking status, BMI, family PIR, drinking status, hyperlipemia and hypertension.

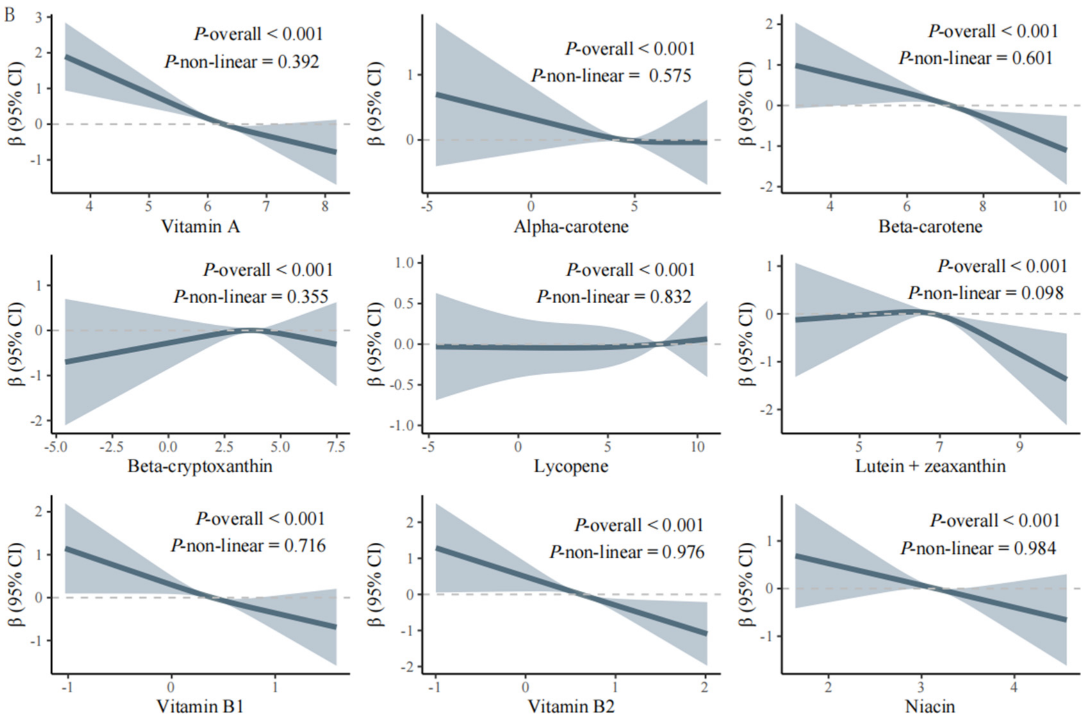

Figure S2 Dose-response relationships between dietary nutrient intake and accelerated aging (B)

The dose-response relationships between dietary nutrient intake (including vitamin A, alpha-carotene, beta-carotene, beta-cryptoxanthin, lycopene, lutein + zeaxanthin, vitamin B1, vitamin B2, niacin) and acceleration of aging (fully adjusted RCS Model). Dietary nutrient intakes were log-transformed. Adjustments in the model accounted for the following variables: age, sex, ethnicity, educational level, exercise, diabetes, history of disease, smoking status, BMI, family PIR, drinking status, hyperlipemia and hypertension.

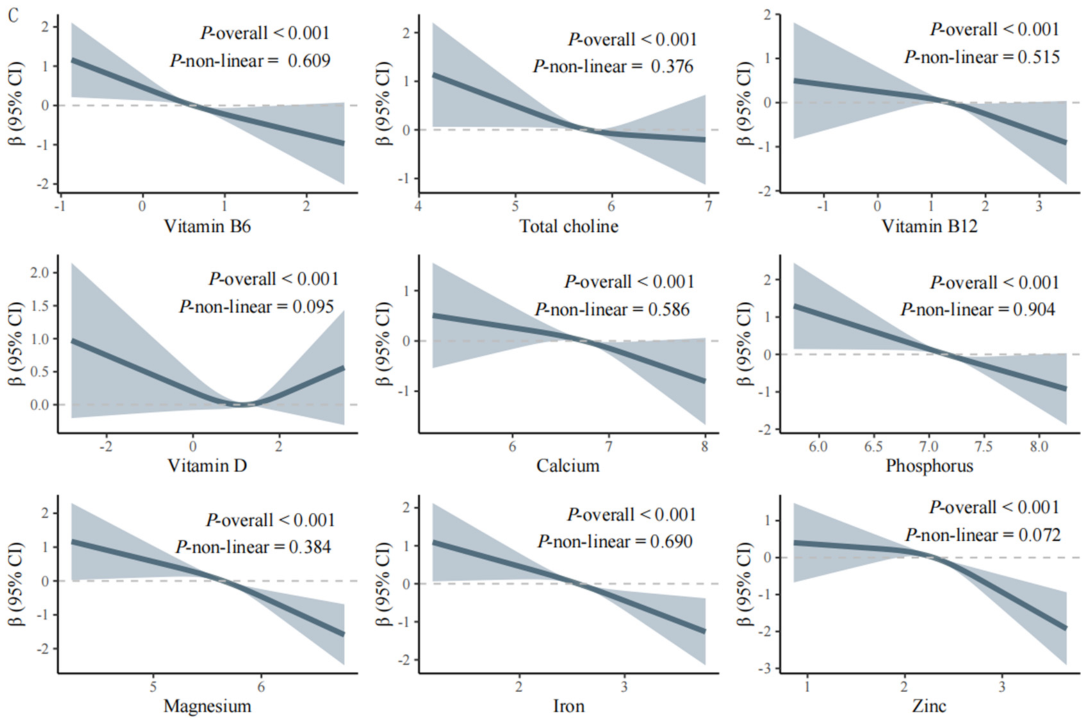

Figure S3 Dose-response relationships between dietary nutrient intake and accelerated aging (C)

The dose-response relationships between dietary nutrient intake (including vitamin B6, total choline, vitamin B12, vitamin D, calcium, phosphorus, magnesium, iron, zinc) and acceleration of aging (fully adjusted RCS Model).

Dietary nutrient intakes were log-transformed. Adjustments in the model accounted for the following variables: age, sex, ethnicity, educational level, exercise, diabetes, history of disease, smoking status, BMI, family PIR, drinking status, hyperlipemia and hypertension.

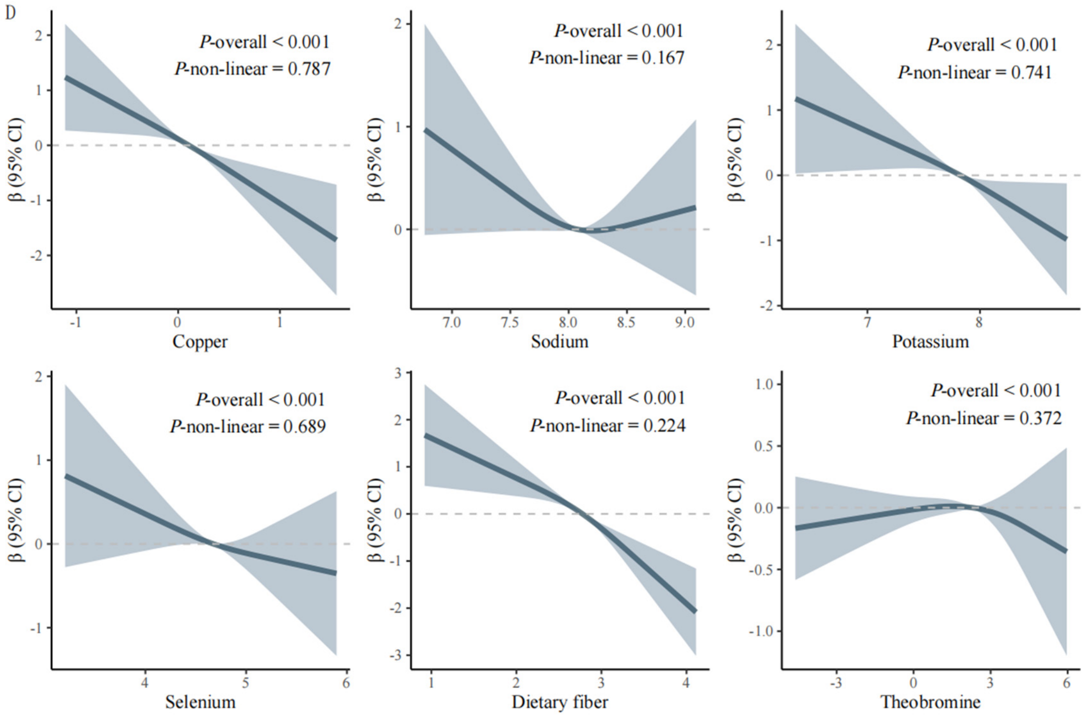

Figure S4 Dose-response relationships between dietary nutrient intake and accelerated aging (D)

The dose-response relationships between dietary nutrient intake (including copper, sodium, potassium, selenium, dietary fiber, theobromine) and acceleration of aging (fully adjusted RCS Model).

Dietary nutrient intakes were log-transformed. Adjustments in the model accounted for the following variables: age, sex, ethnicity, educational level, exercise, diabetes, history of disease, smoking status, BMI, family PIR, drinking status, hyperlipemia and hypertension.

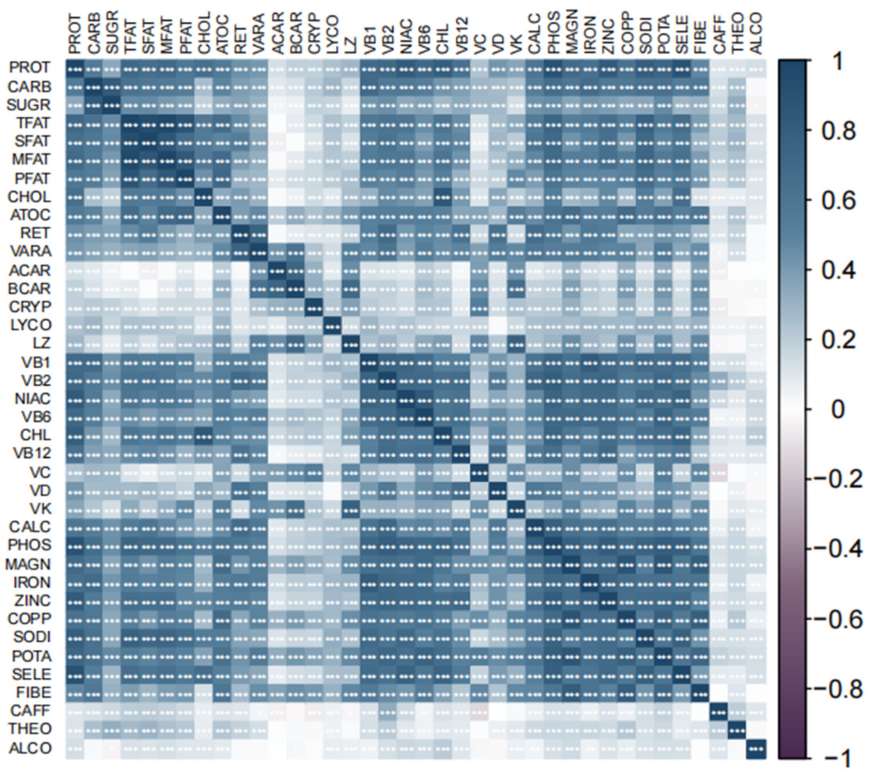

Figure S5 Spearman's correlation matrix among Log-transformed dietary nutrient intakes in the study population

Abbreviations: ALCO: alcohol, BCAR: beta-carotene, COPP: copper, ATOC: Vitamin E, PFAT: Total polyunsaturated fatty acids, THEO: Theobromine, ACAR: Vitamin A, VK: Vitamin K, MAGN: Magnesium, CAFF: Caffeine, SELE: Selenium, FIBE: Dietary fiber, ZINC: Zinc, SODI: Sodium, VC: Vitamin C, NIAC: Niacin, LZ: Lutein + zeaxanthin, IRON: Iron, PROT: protein, VD: Vitamin D, VARA: Vitamin A, LYCO: Lycopene, SUGR: Total sugars, MFAT: Total monounsaturated fatty acids, VB2: Vitamin B2, CARB: Carbohydrate, CHL: Total choline, CRYP: Beta-cryptoxanthin, RET: Retinol, VB1: Vitamin B1, VB12: Vitamin B12, CALC: Calcium, VB6: Vitamin B6, TFAT: Total fat, CHOL: Cholesterol, PHOS: Phosphorus, POTA: Potassium, SFAT: Total saturated fatty acids.

***$P < 0.001$; **$P < 0.01$; *$P < 0.05$
